# Abstracts and Gleanings

**Published:** 1886-09-20

**Authors:** 


### ABSTRACTS AND GLEANINGS.

**Marvelous Results of a New Anæsthetic.**—A week ago a little bald-headed dentist, who lives in Brooklyn, sent invitations to the eminent doctors in this city and Philadelphia, asking them to be present at a series of experiments that he proposed with a new anæsthetic he had discovered. He explained that his compound was something entirely unknown hitherto, and the result of five years' work. He pleaded that he was poor, and could not afford to give his formula to the world; and, although he was willing to demonstrate the uses of his discovery, he preferred to keep its ingredients to himself.

When he appeared in the laboratory of a friend, to demonstrate the practical advantage of his discovery with patients, he found nobody there to listen to him. But on Tuesday afternoon six well known physicians and a reporter, in the parlor of an uptown dental establishment, awaited his arrival. He came at last, mopping his bald head and shaking the rain-drops from his threadbare coat. In a rambling sort of way, while he opened his instrument bag and evaporating pans, the dentist made a little speech, in which he told the story of his discovery.

"For many years," he said, "I have thought that progressive science should devise means of producing natural sleep at will. Knowing that the agents heretofore used for producing anæsthesia seriously interfered with the natural and indispensable functions of the heart, lungs and brain, I therefore determined to investigate and discover, if possible, some agent that would produce natural sleep at will, without pain or danger, or in anyway interfering with the normal organic functions of the human system. In my researches I find the function of the brain suspended in two ways —first, a complete suspension of the brain function, as in coma; second, as in syncope or prolonged faint. No person can be wakeful with a diminution of the blood circulation of the brain. Sleep depends entirely upon that. If, however, the condition of sleep becomes abnormal, and remains so for a considerable length of time, the syncopic effect must produce death. Chloroform, ether, nitrous oxide gas, produced sleep analogous to that existing in coma, and may end in death."

Here the little dentist went into a discussion on the functions of the nerves, and the effect of the old anæsthetics on the system, and their tendency to produce nausea, headache, prostration, and perhaps death. He wound up by saying that his discovery produced natural sleep almost instantaneously, and the patients recovered of their own accord, invigorated and refreshed. He hobbled into a side room and soon appeared with a decrepit old woman. He promised to remove all the decayed stumps from her mouth if she allowed him to use his anæsthetic in the operation. He saturated a napkin with a substance that looked like water, and emitted a pleasant odor. For an instant he held it over the old lady's nose, telling her to breathe freely, and in thirty seconds she was unconscious. He allowed the napkin to remain, and for over two minutes he worked, extracting sixteen teeth. The patient never moved, and to all appearances she did not feel the pain attending the operation. On removing the napkin, almost immediately she revived, and stepped out of the chair, as fresh and hearty as when she got into it.

She said that she had a vague idea of what was going on while under the influence of the anæsthetic, could not move hand or foot. She was reminded of a person in a trance, and described the symptoms she experienced as a sort of suspended animation. She said that she knew that the dentist was pulling her teeth out, but she felt no pain.

Eight persons were made and kept unconscious from a half to two and a half minutes. In one case the napkin was allowed to remain for six minutes. The patients told stories similar to the old lady's, and went away in good spirits. The reporter was the last subject, and these, in brief, were the sensations he experienced:

From the moment the napkin was placed over his face a feeling of unconsciousness came over him, and at the end of the third free inspiration everything became blank. His arms hung at his side, and he could feel that they were there, but they were beyond the voluntary control of the will; a sense of dreamy languor followed, and, as if on the wings of a fleet bird, he was borne through the air high above the earth. The sensation was altogether pleasant. Then the scene changed, and his skull tingled as if a million minute hammers, no bigger than a fine needle, began pounding all at once. They shattered into fragments in an instant, the napkin was removed, and all was over. Recovery was instantaneous, and all effect was gone. The time, taken by one of the physicians, was one and three-quarters minutes.

The little dentist was urged to reveal the component parts of his anæsthetic, but he refused, saying that he was too poor, and as much as he would like to do something for science, he had to think of his hungry wife and children at home. The physicians who witnessed the experiments said the results were marvelous, but as long as the whole thing could not be scientifically explained, to the profession at large, they would never accept it. Perhaps it will never receive the just recognition it so richly deserves.—*New York Star.*

**The Local Use of Liquid Ergot, Normal, in Chronic Gonorrhœa.**—Dr. Harvey Craig, Mansfied, Ohio, in *Medical Age:* Hearing of the success of Dr. N. V. Speere, of Quincy, Ohio, in the treatment of gonorrhœa with local applications of normal ergot (such as that prepared by Parke, Davis & Co., of Detroit), and realizing the need of some more satisfactory remedy in the treatment of this disease, in its many stages, I resolved to see if the same good results would follow in my own practice, and therefore vowed that the next case of chronic gonorrhœa that came under my care should have the benefit of the experiment. In a day or two the opportunity presented itself.

CASE I.

A young man, a salesman in one of our large manufacturing establishments, had suffered long and suffered much. He had tried all the "patents," "Big C.'s," etc., to be had, and had also received "rational" treatment from one of our regular physicians. All this availed him nothing. After hearing his story, and knowing him to be a young man of temperate habits, never indulging in alcoholic stimulants, and observing a proper diet for so long a time, "my heart almost failed me." I concluded his was a very bad case. Nevertheless, I grimly determined to do what I could, recognizing the fact that success is not without effort.

First, I introduced a large sized bougie, finding no difficulty in doing so. He complained, however, of pain, as the instrument was passing. After satisfying myself that no stricture was present, I gave him a small vial of normal liquid ergot, instructing him to use for an injection one part of this to four parts of water (distilled), once daily for a week, and at the end of that time (or sooner if he chose) to report progress. Did not see him again for about ten days, at which time he called to report. He very abruptly exclaimed: "What the h—— was that you gave me?" I made some evasive reply, as I feared my treatment had proved disastrous in some way. Finally he said: "Well, I spent over $35.00 on this business before I came to you, and this little vial cured me up." He remarked after the first application he could notice an improvement, and at the end of the fith day he was entirely free from discharge and pain.

CASE II.

Thos. W——, single; cabinet maker; had contracted gonorrhœa about three years ago. Had received rational treatment, but was not benefitted. He came to me for treatment about the last of May, 1886. This was a very obstinate case, but finally yielded to treatment and was discharged cured, July 30, 1886.

CASE IX.

John H——, a moulder; single; had had gonorrhœa about one year. This case was very much like case one, except that the patient was intemperate. He was kept under treatment for about four weeks, owing to the fact that he would get drunk. This retarded the cure in his case.

Cases three, four, five, six, seven, eight and ten yielded readily to treatment, and were discharged cured within from six to nine days.

Since then I have used it repeatedly and with the same good results. I have a little army here that would, if they knew upon whom to shower their blessings, bless the man that made the experiment of using local applications of normal liquid ergot in chronic gonorrhœa.—*Medical Age.*

**The Preventive Treatment of Syphilis.**—Dr. Charles E. Jennings thus writes to the Lancet, July 17: The following case is of interest, as showing how syphilis may be prevented by prompt local and general treatment before the chancre has arrived at maturity:

Early in November, 1885, Mr. A. B. C—— consulted me for an eczematous affection of the skin, which itched intensely, associated with a few papules and vesicles, the eruption being sparsely scattered over the loins, buttocks, back, and upper arms. An aperient was prescribed, and glycerole of lead applications were ordered, but on November 11 the disease remained in *statu quo.* The aspect of the dermatitis, in part, suggested scabies, but the integument was healthy at those points which are specially prone to become the seat of scabies, viz, the clefts between the fingers and the flexures of the wrists. Moreover, the sarcoptes hominis was not detected miscroscopically, after careful search in the ordinary manner. The case was further complicated by the recent appearance of a hard, elevated nodule, the size of half a pea, with an inflammatory zone around, situated on the dorsum of the penis immediately behind the corona in the median line. There was a history of impure contact, and nearly three weeks previously the covering of the penis had become abraded during coitus at the precise spot where this small nodule had developed during the past three or four days. The lymphatic glands in both inguinal regions were slightly enlarged. The physical condition of the lesion on the penis led me to consider it a hard chancre not yet arrived at maturity, and, being immature, the prompt destruction of the chancre might arrest the elaboration of the syphilitic virus at the point of inoculation, and its absorption therefrom. Accordingly, the chancre and surrounding parts were freely painted with a solution of hydrochlorate of cocaine, to ensure anæsthesia, and the chancre was destroyed with Paquelin's cautery. I suggested that Sir William Jenner's opinion should be sought with reference to the skin affection. On November 12th, Sir William considered the dermatitis due to scabies, and that its origin around the loins and buttocks was caused by contagion implanted during congress coincident with the receipt of the syphilitic virus. He suggested the application of sulphur ointment to the affected skin, and thought that the free exhibition of mercury internally was also advisable in order to combat the effects of any portion of syphilitic virus already absorbed. This treatment was fully carried out, and the dermatitis quickly disappeared. The sore on the penis healed under iodoform dressings. The internal use of mercury was discontinued after six weeks, the patient soon regaining his usual health, and none of the secondary manifestations of syphilis have appeared. The success which followed the destruction of the chancre in this case by Paquelin's cautery, with the simultaneous employment of mercury, leads me to strongly recommend this course for syphilis, before the chancre has arrived at maturity, in preference to the opposite plan adopted by so many, viz, that of waiting for the appearance of secondary manifestations before commencing anti-syphilitic treatment. The use of cocaine is invaluable as rendering the caustic application painless.—*Med. and Surg. Reporter.*

**Removal of Foreign Bodies from the Ear.**—In the British Medical Journal Dr. J. H. Granshaw suggests the following method for removing foreign bodies from the ear :

A large syringe, holding four or six ounces, a basin of rain-water soap-suds as hot as can be borne, and a steady hand, are all that is required. With this simple apparatus, I have, over and over again, removed cherry-stones, beads, buttons, slate-pencils, etc., from the ears of children, and always without pain; nor has it ever failed me. The injection of a few syringefuls will generally suffice.—*Ibid.*

**Ethoxycaffeine in the Treatment of Migraine.**—M. Dujardin-Beaumetz uses the following formula:

| | |
|---|---|
| Ethoxycaffeine.......... .......... | aa.....4 grains. |
| Salicylate of sodium................ | |

To be taken in a single dose as soon as the pain begins to be felt By adding a sixth of a grain of hydrochlorate of cocaine, gastric irritation will be prevented.—*N. Y. Med. Jour.*

**On the Statistics of 3036 Cases of Labor, by Hiram Corson, M. D.**—Dr. Wm. Goodell read the paper, which will be published in full in the *New York Medical Journal.*

In the Transactions of the Medical Society of the State of Pennsylvania for 1863 may be found an article headed "Midwifery in the Country," in which are drawn statistics from 2387 consecutive cases of labor, to which are now added 649 cases, making in all 3036 cases, with 3087 children. Head presentations (vertex), 3012; breech, including knees and feet, 58; shoulder and arm, 5; face, 12. Twins in 51 cases. Ergot was used in 139 cases in first series. Forceps were used 28 times in the first 2387 cases, and 31 times in the last 649 cases. Version was performed twice in the last series. One primiparæ was 52 years of age. Puerperal convulsions in eight cases, all recovered. There was a total of 190 cases, in which the children were born before the doctor arrived, and in which the mothers did well under nature's management, and were saved the fright and suffering which, if Crede had been present, would have resulted from his fears of hemorrhage, his rush of one hand into the vagina the moment after the child was born, and the grasping, squeezing, and forcing down of the womb by the other hand on the tender, sore abdomen, to say nothing of having that heavy hand pressing on a tight bandage for two hours more, in accordance with regulation orders.

In the practice of this art I have not followed the requirements of the times. I have considered labor a natural process, and that my duty consisted in awaiting the action of the patient's forces; not setting them aside and myself usurping the duties which the natural efforts would have achieved without difficulty, but coming to my patient's aid only when her forces seemed inadequate to the performance of their duties.

I have learned that the forceps are used very often, many times oftener in proportion to number of cases than twenty years ago, and that this is done in the early part of labor, not because nature is inadequate to the work, but because as the physician had never hurt *himself* by using the instrument, and wished to get away speedily, as he had other patients who needed attention; and though the condition of the lying-in woman would well have permitted him to visit his other patients and return in time to aid her if she needed aid, still he would not do it, through the fear that the child might be born in his absence, or some other doctor be called in his place. The graduate of the month can now use forceps and all the other swift-sure means of speedy delivery without hurting his hands or spraining his back. Of course the sufferings and fate of the woman were of secondary importance. Being anxious to learn all about the advances in midwifery, I attended the meeting of the American Gynæcological Association in Philadelphia, a few years ago. There I heard from the mouth of more than one eminent gynæcologyist—men greater than mid-wives: "Every year I use the forceps more frequently than before." There, too, I was amazed to learn from the experience of these speakers, how numerous were the cases of lacerations of the cervix and perineum; so numerous, indeed, that when coupled with the fact that the advocates of frequent use of the forceps were teachers of midwifery, and also eminent surgeons skillful to "repair" these lacerations, and that these repairs brought large remuneration to the surgeons, I was dazed—I knew not what to say. I could not believe it possible that the earnest gentlemen before me could have conspired to teach this rushing plan of delivery in order that lacerations should be produced, so that the business of repairing should be brisk and profitable. It was pleasant, a few months later, to hear that the eminent gynæcologist, Dr. Goodell, attributed the great increase of lacerations to the use of the forceps, and earnestly denounced their indiscriminate employment.

In October, 1880, the *Boston Medical and Surgical Journal* contained an article by J. W. Elliott, M. D., on "Antiseptics in Gynæcology," with full directions for their use in obstetrics, to prevent puerperal fever by destroying poison-germs which might be introduced by the doctor or nurse. "At the beginning of labor the patient should have a hip-bath, the hair should be cut away from the genitals, the vagina and vulva should be washed with soap and disinfected with carbonic acid. During labor every examination should be preceded by a vaginal injection of a 3 per cent. carbolic solution, to prevent the examining finger carrying germs lodged at the vulva or in the vagina up to the uterus (which is about to be more or less lacerated). After delivery, the uterus and vagina should be considered as a deep and important wound, which may heal by first intention, or in which the secretions may stagnate, become putrid, and be absorbed."

As I had never before heard of Dr. Elliott, I went on in my usual way, taking no razor to shave the parts, no syringe, no carbolic acid, but let nature go on with her work, pleased to see how steadily and perfectly she accomplished it. Dr. Corson asks: Is labor a natural process? Are antiseptic solutions when thrown into the uterus and vagina safe? and answers these questions from the standpoint of his own experience, and by quotations from

Albert H. Smith's lecture "On the Relation of Cleanliness to the Prevention of Puerperal Septicæmia;" Dr. W. O. Stillman's account of the precautions taken by Carl Braun, of Vienna, to prevent infection to the lying-in woman; Dr. T. G. Thomas, of New York, a paper read before the New York Academy; Dr. George J. Harrison, in reply to Dr. Thomas; and to papers by Fritsch and Kustner, and gives his own experience in his first labor case in 1827.

Dr. Corson next considers the forced delivery of the placenta, and claims the originality of the Crede method in its principal details for Professors James and Dewees, the latter saying that it had been long since recommended by Monsieur Darse, of Paris. He criticises the unnecessary severity of the latter part of the Crede method, and much prefers the directions by Prof. Penrose, and then details his own method, which leaves more to natural powers, giving morphia if rigid contractions of the os occur before the placenta comes away.

He recommends venesection to the extent of 10 or 20 ounces, to relax a rigidly contracted os in the first stage of labor. If this is not immediately successful, he gives morphia internally. He uses ether to relax when pains are too severe.

Dr. Corson next considers the question of tying the cord. Should it be tied at all? How soon after birth should it be divided? What is gained by waiting until pulsation ceases in the cord? These are questions to which careful consideration has been given, and which are fully answered. Dr. Corson does not use a binder; has not done so for twenty years. He does not think that it prevents relaxation of the uterus, but that it favors prolapse of that organ. He gives his own experience, and fortifies it by that of several other physicians. He thinks the hasty extraction of the placenta, the compression of the uterus by the hand, and the application of the binder with the avowed intention of preventing hemorrhage, have a bad mental effect on the patient, and predisposes to the very trouble we are seeking to avoid. He gives several instances of mental influences in stopping hemorrhage. He has never used hot water, vinegar, lemon or ice into the uterus for flooding, but has applied ice externally.

Puerperal convulsions, ten cases all recovered. His treatment consists largely in free venesection, cold water poured over the head, and morphia internally. He recommends the hand-book of Dr. Ezra Michener for the successful treatment of this malady. He would also avert convulsions by bleeding before labor to relieve headaches, if accompanied by congestion of the face. Of puerperal or septicæmic fever he knows nothing, having never seen it.—*Medical and Surgical Reporter.*

**Therapeutic Value of Coca Preparations in Children.**—In a paper read before the association of German physicians and philosophers in Strasburg, Dr. Richard, pathologist, of Halle, presented the following conclusions from his experience in the use of coca with children:

The tincture of coca, in doses of from 5 to 20 drops every two hours, according to the age of the child, has proven of advantage in stomach and bowel diseases; and even in doubtful cases of cholera nostras the vomiting and purging cease after from 50 to 100 drops have been given. The diet is not to be neglected. Doubtless in these cases the alcohol contained in the tincture is to be credited with part of the success.

In diseases of the respiratory organs tincture of coca is without influence.

The extract of coca, given in conditions of spasm not caused by anatomical derangements of the nerve centers, as in hysteria and epilepsy, is in some cases of advantage and in others of none.

The extract of coca is more active than the tincture. After the use of the extract children complain of dullness and a peculiar feeling about the head, with costiveness.

Great service was rendered by penciling the pharynx with a 5- to 10-per cent. solution of cocaine in different forms of angina. Pain and difficulty of swallowing ceased, and reflex irritability is considerably lowered. In whooping cough remarkable success was obtained, where penciling the pharynx three times daily with a 5-per cent. solution has been sufficient to reduce paroxysms coming on twenty times a day to three or four in the twenty-four hours.—*American Prac. and News.*

**Fatal Results from "Splitting the Cervix."**—Dividing the cervix at the external, or at the internal os, or in the intervening portion, though not long since a comparatively frequent operation for dysmenorrhœa or sterility, is now rarely done. Most operators now turn to dilators for the treatment of cases where incision was formerly done; one wing of the army of gynæcologists still fights under the banner of mechanical uterine pathology, only in place of hysterotomes, its enthusiastic soldiers use dilators. Possibly it is only a question of time when many of the dilators will be placed in the grave beside the hysterotomes, if the teaching of men like Duncan, Schultze, and Williams prevails, and the mechanical theory of uterine disease is cast aside.

However this may be, we have been somewhat astonished to know of the mortality which Sims had from this operation. Pajot states, in a recent lecture, that he knew of at least four deaths of women upon whom Sims had performed his operation of division of the cervix, and he believes that other similar accidents happened to him. In the light of these facts, the profession is to be congratulated upon the fact that the operation has fallen into disuse.—*Medical News.*

**Water as a Diuretic.**—Dr. Brunton says (in the Practitioner), that water is, perhaps, the most powerful diuretic we possess, although fewer experiments have been made with it upon animals than with the others. The diuretic action of water drunk by a healthy man is very marked, and it appears impossible to explain its elimination by a mere increase in blood-pressure, whether general or local. It has the power of increasing tissue-change, and thus multiplying the products of tissue-waste which result from it, but it removes these waste products as fast as they are formed, and thus, by giving rise to increased appetite, provides fresh nutriment for the tissues, and acts as a true tonic. In persons who are accustomed to take too little water, the products of tissue waste may be formed faster than they are removed, and thus accumulating may give rise to disease. Many gouty persons are accustomed to take little or no water, except in the form of a small cup of tea or coffee, daily, besides what they get in the form of wine or beer. A large tumbler of water drank every morning, and especially with the addition of some nitrate or carbonate of potassium, will prevent a gouty paroxysm. Still more numerous, possibly, in the class of people who arise in the morning feeling weak and languid. Many such people are well fed, they sleep soundly, and it seems almost impossible to believe that the fatigue which they feel in the morning can result from imperfect nutrition, more especially as one finds that after moving about the languor appears in a great measure to pass off. It seems that this languor must depend upon imperfect removal of the waste products from the body, as we know that the secretion of urine in healthy persons is generally much less during the night than during the day. Such people should drink a tumbler of water before going to bed, in order to aid the secretion of urine and of the waste products during the night.—*Prac. and News.*

**Permanganate Potassium in Snake Bites.**—Dr. J. Berger reports the following case in the St. Louis *Medical Journal* for July 1886: I would respectfully call the attention of the readers of the *Joural* to the new antidote for snake poison, permanganate potassium. For a full history of the discovery I refer the reader to the *Therapeutic Gazette*, Vol. V., May, 1884, p. 212. When I read the article referred to above, it made such an impression on my mind that I determined to give it a test the very first opportunity. In August, 1884, I was sitting on the varanda reading, when I heard my son, Frank, æt. fourteen, say he was snake bitten. I at once made a solution of permanganate of potassium, and filled my hypodermic syringe. By the time he had killed the snake and got to where I was, I was ready for business. He was bitten on the thigh by a large copperhead, the fangs entering the flesh about half an inch apart. The wound was bleeding some and had commenced to swell, the swelling having extended in every direction from the bite about an inch, and was causing considerable pain. I introduced the needle of the syringe under the wound and injected 10 minims of the solution, waited a few minutes and repeated the injection. In about fifteen minutes repeated the injection again. After the first injection the swelling all disappeared and the pain ceased as soon as the smarting from the effect of the medicine was over, and the only inconvenience that was ever felt after was a small abscess that formed where the needle was introduced.

There is only one drawback to this remedy, and that is it must be used in a few minutes after the wound is inflicted to be entirely effectual.

I hope this remedy will soon become generally known and adopted as the treatment for snake bites, as it would do away with that unscientific, injurious, death-dealing practice of filling every person full of bad corn-whisky and alcohol, that is bitten by a snake. —*Medical Age.*

**Puncture of the Nerve Sheath in Sciatica.**—Sir Joseph Fayrer. M. D., in *Practitioner:* Some years ago I was asked by a medical man in Calcutta to see a case of aggravated sciatica of long standing in a man of middle age. The pain was very severe, continuous, and liable to increase of a paroxysmal character. The posterior muscles of the thigh were somewhat atrophied; the patient himself was wasted and worn by continued suffering and deprivation of rest and sleep. There was no history of syphilis, gout, rheumatism, or other specific cause. A malarial origin of course was possible, but as far as I can remember, there was no satisfactory explanation of the origin of the disease. All the usual methods of treatment had been resorted to, but without relief. On examining the limb more carefully, I detected some feeling of fluctuation; a fullness and tenderness in the course of the sciatic nerve near its origin, in the upper part of the limb; I thereon introduced a long narrow knife into the swelling, until it entered the sheath of the sciatic nerve; this gave exit to a certain quantity —a couple of drachms or so—of clear serous fluid, which was followed by immediate relief, and rapidly resulted in complete recovery. I have seen other cases, none so well marked as this one, however, and with much less effusion of fluid, where incision, or rather I should say puncture, has given relief; but I am not aware if others have had similar experience, and would call attention to it as of practical interest. It has long been known that acupuncture with needles not infrequently gives great and permanent relief in some forms of deep-seated pain; indeed it is practiced by some Oriental races for this purpose. I think it possible that its success may sometimes be due to the relief of tension, caused by evacuation of the fluid which has accumulated as a result of inflammation, in large or small nerve-trunks.

**Salicylate of Cocaine in Asthma.**—A comparatively new method of treatment in asthma nervosum has lately been tried by Professor Mosler of Greifswald. It is now well known that cocaine has not only a local action on the sensory nerve-endings, but also a central one, which, at first stimulating to the nerve-centers, may, if the drug be pushed, become sedative or even narcotic. By this peripheral and central effect, it may, therefore, act in such spasmodic diseases as asthma. Early last year, Beschorner published two cases of this disease which were much benefitted by cocaine. In three cases Professor Mosler has obtained excellent results. All these cases occurred in young people of 23 to 25 years of age, and were uncomplicated by any organic heart or lung disease. The drug was given snbcutaneously, in doses of 0.4 gramme, at the commencement of the attack. The first patient, who had a bad family history as regards lung-disease, and in whom the asthma had lasted 11 years, was relieved after the third injection; two more doses caused abeyance of the attacks (which occurred previously every day) for a fortnight, when the patient was lost sight of. In the second and third cases, the treatment was more rapid in cutting short the attacks, which in the end were postponed for three or four weeks during which the patients continued under observation. The injections caused, in one case, a slight sense of faintness and the appearance of dark spots before the eyes; but these symptoms soon vanished. It is, of course, impossible to draw from these cases any conclusions as to the permanent benefit of the treatment. Extended experience will, perhaps, show that the drug is only a palliative. It is in the hope of inducing other practitioners to try the treatment that Professor Mosler has published the results of his cases —*London Lancet.*

**The Use of Hot Water in some of the Corneal and Conjunctival Inflammations** was the title of a paper by Dr. B. E. Fryer, of Kansas City. In this plan of treatment water was at as high a temperature as could be borne. After a few hours, a temperature of 140° F. could not be tolerated. The water should not be cooler than this, but as much hotter as the patient could endure. A method of using it was by fomentation with a napkin dipped into the hot water and (not wrung out) applied to the closed eyelids. This was continued for half an hour at a time and repeated every one, two, or three hours, day and night. It might also be applied by suspending a vessel above the patient and allowing the water to escape through a tube, thus keeping up a continuous action. In some cases the temperature might be raised, almost to the boiling point. During the intervals between the applications, a cloth wrung out of hot water was allowed to remain over the eyes. In some cases of purulent ophthalmia the hot water might be thrown into the conjunctival sac. In purulent conjunctivitis this application cut short the attack more quickly and safely than the use of ice-cold water. In gonorrhœal ophthalmia it quickly lessened the swelling and diminished the tendency to ulceration of the cornea. If ulceration had begun, it was less likely to progress and the amount of cicatricial tissue was lessened. In these cases the author occasionally used instillations of sublimate solution or finely powdered. iodoform. In catarrhal and phlyctenular ophthalmia it was a good adjuvant. In acute and chronic keratitis it was useful. Its most marked effects were seen in cases of corneal ulcer. The small amount of opaque tissue left was astonishing. The pain and photobia were also diminished.—*New York Medical Journal.*

**Turpine in Chronic Bronchitis.**—Turpine, which has neither smell or taste, can be given at a dose of from one to three grammes daily. It diminishes the expectoration in chronic brochitis, but has hardly any effect in phthisis. Its action is the same as that of turpentine and creasote, but it is less disagreeable to take, and therefore preferable in some cases.—*London Medical Record.*

